# Genkwadaphnin Induces IFN-γ via PKD1/NF-κB/STAT1 Dependent Pathway in NK-92 Cells

**DOI:** 10.1371/journal.pone.0115146

**Published:** 2014-12-17

**Authors:** Ho-Bum Kang, Kyung-Seop Ahn, Sei-Ryang Oh, Jae Wha Kim

**Affiliations:** 1 Medical Genomics Research Center, Korea Research Institute of Bioscience and Biotechnology, Daejeon, Republic of Korea; 2 Immune Modulator Research Center, Korea Research Institute of Bioscience and Biotechnology, 685-1 Yangchung-ri, Ochang-eup, Cheongwon-gun, Chungbuk, Republic of Korea; University of Pittsburgh School of Medicine, United States of America

## Abstract

The flower buds of *Daphne genkwa* Sieb. et Zucc. have been used as a traditional Chinese medicine although their functional mechanisms have not been discovered yet. We have studied the potential effects of the plant extracts on natural killer (NK) cell activation, and isolated an active fraction. Genkwadaphnin (GD-1) displayed a potent efficacy to induce IFN-γ transcription in NK cells with concentration- and time-dependent manners. GD-1 treatment triggered the phosphorylation of PKD1, a member of PKC family, MEK and ERK, resulting in IKK activation to induce IκB degradation, and the nuclear localization of p65, an NF-κB subunit, which regulates IFN-γ transcription. GD-1 effect on IFN-γ production was blocked by the addition of Rottlerin, a PKC inhibitor, CID 755673, a PKD inhibitor, or Bay11-7082, an IKKα inhibitor. The nuclear localization of p65 was also inhibited by the kinase inhibitors. Secreted IFN-γ activates STAT1 phosphorylation as autocrine-loops to sustain its secretion. GD-1 induced the phosphorylation of STAT1 probably through the increase of IFN-γ. STAT1 inhibitor also abrogated the sustained IFN-γ secretion. These results suggest that GD-1 is involved in the activation of PKD1 and/or ERK pathway, which activate NK-κB triggering IFN-γ production. As positive feedback loops, secreted IFN-γ activates STAT1 and elongates its production in NK-92 cells.

## Introduction

The flower buds of *Daphne genkwa* Siebold et Zuccarini (Thymelaeaceae) is a traditional Chinese toxic herb, which is commonly used for diuretic, antitussive, expectorant, edema, and asthma treatments although their specific biological activities have not been defined yet. The medicine also showed anti-cancer effects on malignant ascites and solid tumors [1]–[3]. Daphnane diterpene esters, genkwadaphnin (GD-1) and yuanhuacine, have been isolated from the dried flower buds to possess significant anti-tumor activities via the suppression of DNA synthesis and the activation of apoptotic pathways against leukemic cell lines [4], [5]. In addition, yuanhuacine has showed cytotoxic activities against solid tumor cell lines like MCF-7 and Colo 205 [6].

Natural killer (NK) cells develop primarily in the bone marrow, thymus and lymph nodes, and are distributed in many organs throughout the body circulating through the blood on patrol for the presence of transformed or pathogen-infected cells. However, there are increasing evidences that NK cells include distinct subset populations with discrete functions according to their developmental origin and locations. For example, human NK cells can be divided into two sub-populations based on the expression of surface receptors: CD56 and CD16 [7], [8]. CD56^dim^CD16^+^ NK cells occupy the majority of blood and spleen NK cells, which are highly cytotoxic but have low levels of cytokine secretion. In contrast, most NK cells in the lymph node are CD56^bright^CD16^−^ with poor cytotoxic capability but produce large amount of proinflammatory cytokines such as IFN-γ, TNF, and GM-CSF. CD56^dim^CD16^+^ NK cells also secrete IFN-γ after activation. The ability to secrete IFN-γ made NK cells as a regulator of the coordinated activation of innate and adaptive immunity.

NK and NKT cells constitutively express IFN-γ mRNA, which allows for the rapid induction and secretion of IFN-γ on infection. IFN-γ is also produced by a wide variety of cells in response to the presence of double-stranded RNA, a key indicator of viral infection [9], [10]. The IFN-γ produced by activated immune cells assists the immune response by inhibiting viral replication within host cells, activating NK cells [11], increasing antigen presentation to lymphocytes [12], and inducing host cell resistance to viral infection [13]. IFN-γ production is controlled by cytokines secreted by antigen-presenting cells (APCs), most notably interleukin (IL)-12 and IL-18. These cytokines serve as a bridge which links infection with IFN-γ production in the innate immune response [14]–[20]. IFN-γ is also involved in the control of tumor initiation, growth, and metastasis [21]–[23]. IFN-γ directly enhances the immunogenicity of tumor cells and stimulates the immune response against transformed cells. Thus, the induction, duration, and amount of IFN-γ produced must be both closely controlled and delicately balanced for optimum host wellness [24]. IFN-γ orchestrates leukocyte attraction and directs the growth, maturation, and differentiation of many type of cells [25]–[27] in addition to enhancing NK cell activity [28].

The main pathway for IFN-γ production in IL-12-induced NK cells is dependent on the activation of PKCθ [29]. Tassi et al. [30] reported that the engagement of NK-cell receptors signaling through ITAMs results in prompt activation of PKCθ, which is a member of the PKCs family. Analyses of NK cells from PKCθ–deficient mice indicated that PKCθ is absolutely required for ITAM-mediated IFN-γ secretion [30]. PLCγ is also a fundamental intrinsic factor for IFN-γ secretion. The basal level of IFN-γ production was significantly reduced in PLCγ2-deficient NK cells, and, in contrast to WT cells, stimulation with anti-NK1.1 did not induce the augmentation of IFN-γ release [31]. The PLCγ2-deficient NK cells were severely impaired in their ability to produce either IFN-γ or GM-CSF,http://www.jimmunol.org/cgi/content/full/177/8/5365 - F5#F5 and PLCγ2 is known to play a critical role in NKG2D as well as NK1.1-mediated cytokine production [32]. In the mouse, these include the γ-chain of FcRs (FcRγ), receptors that are involved in tyrosine phosphorylation of the associated adaptor ITAMs, which in turn recruit the protein tyrosine kinase Syk. This kinase activates multiple downstream signaling mediators, including linker for activation of T cells, 76 kDa, PLCγ, PI3K, and the ERK kinases. Collectively, these signaling mediators trigger gene transcription and the cellular programs for exocytosis of the lytic granules that allow NK cells to lyse target cells and the production of proinflammatory chemokines and cytokines, particularly IFN-γ [33].

Previously, we have reported the isolation of two Daphnane diterpene esters, GD-1 and yuanhuacine, from the flower buds of *Daphne genkwa*, and their tumor suppressive effects on a human myelocytic HL-60 cells and the inoculated Lewis lung carcinoma [34]. In this study, the effect of the flower buds on NK cell activation was discovered by analyzing the secretion of IFN-γ, and the active ingredients were identified to be GD-1 followed by its mechanistic studies. GD-1 has induced the activation of protein kinase D1 (PKD1), a member of protein kinase C (PKC) family, and ERK, followed by the activation of the transcriptional activities of NF-κB and STAT1 resulting in the up-regulation of IFN-γ production.

## Materials and Methods

### Isolation of GD-1

GD-1 was isolated from the dried flower buds of *Daphne genkwa* as previously reported [5]. Briefly, the dried flower buds of *Daphne genkwa* were extracted with MeOH and the methanolic extract was resuspended in water followed by the sequential extraction with *n*-hexane, CHCl_3_ and EtOAc. CHCl_3_-soluble fraction was loaded onto silica gel and eluted using a gradient of CHCl_3_ and MeOH to give 10 fractions. Fraction 3 (F3) was loaded onto an RP C-18 column and eluted with MeOH/H_2_O (7∶3). Among ten fractions (F3-A to J), F3-C was separated by the succession of one more round of silica gel eluted with hexane/EtOAc (15∶1), and RP C-18 eluted with MeOH/H_2_O (3∶1) to give GD-1.

### Cell culture

All human myeloid cells (U937, THP-1, HL-60, K562, and NK-92 cells) were obtained from American Type Culture Collection (ATCC) and cultured in a humidified incubator with 5% CO_2_ at 37°C. The human IL-2 dependent NK lymphoma, NK-92, was maintained in α-MEM (Life Technologies, Karlsruhe, Germany) containing 20% FCS (HyClone, Logan, UT), 2 mM L-glutamate, 100 µg/ml penicillin, 100 µg/ml streptomycin (Life Technologies) and supplemented with 100 U/ml IL-2 (Chiron, Emeryville, CA). U937, THP-1, HL-60 and K562 cells were maintained in RPMI-1640 (Life Technologies) containing 10% FCS (HyClone).

### Enzyme-linked immunosorbent assay (ELISA)

Quantification of human IFN-γ was performed using commercially available mAb pairs (Endogen, Woburn, MA, USA). Cell-free supernatants were collected 18hr after GD-1 treatment at 37°C. In brief, the microtiter plates were coated with a mouse anti-IFN-γ antibody and supernatants were added and incubation for 1 h at 37°C was carried out. Subsequently, monoclonal mouse anti-human IFN-γ (biotinylated, 0.05 µg/ml in blocking buffer, Endogen) was mixed with HRP-conjugated streptavidin and the mixture was then added to the wells for further incubation of 1 h. IFN-γ were detected by measuring the optical density in an ELISA reader (KC junior, BIO-TEK Instruments Inc., VT, USA) at 450 nm with a reference wavelength set at 630 nm. For the detection of IL-2, IL-12, TNFα and IL-10, ELISA kits from Endogen were used following the manufacturer's protocols. Results are shown as the means of triplicate wells ± SEM.

### PKD1, ERK, NF-kB and STAT1 Inhibitor treatment

Rottlerin, an inhibitor of PKC (Calbiochem, La Jolla, CA), CID755673, an inhibitor of PKD (Tocris Biosciences, Ellisville, MO), Bay11-7082 [(E)-3-(4-methylphenylsulfonyl)-2-propenenitrile], an inhibitor of IkB-α phosphorylation (Sigma, St. Louis, MO) and fludarabine, an inhibitor of STAT1 (Santa Cruz Biotechnology, Santa Cruz, CA) were added to the medium at various concentrations between 0 and 100 µM in DMSO (Sigma, St. Louis, MO). DMSO was used as a vehicle control.

### Cell lysis and Western blot analysis

Soluble lysates from the cultured cells were extracted in ice-cold SDS-lysis buffer [50 mM HEPES, 150 mM NaCl, 0.2 mM EDTA, 0.5% NP-40, 0.1% SDS, 1 mM Na_3_VO_4_, 10 mM NaF, and Complete Protein Inhibitor Cocktail (Roche)] for 30 min on ice. Thirty to fifty micrograms of the cell lysate was resolved by SDS-PAGE on 10% or 12% gels and transferred to PVDF membranes (Millipore, Billerica, MA, USA). The membranes were incubated with primary antibodies followed by peroxidase-conjugated anti-rabbit or anti-mouse immunoglobulin (Ig) G secondary antibodies (Calbiochem, EMD Chemicals Inc., San Diego, CA, USA) and ECL reagent (Millipore, Billerica, MA, USA) for band visualization. To verify equal loading and adequate transfer, the membranes were probed with anti-α-GAPDH antibodies (Santa Cruz Biotechnology, Pasadena, CA, USA). The primary antibodies were anti-p50, anti-p65, anti-IFN-γ (Santa Cruz Biotechnology), anti-phospho-PKC isoform, anti-PKC isoforms (Cell signaling Technology).

### RT–PCR

A two-step RT–PCR reaction was performed using reverse transcriptase with oligo-dT primers and M-MLV reverse transcriptase (Promega, Madison, WI, USA) with specific primer pairs. Total RNA was isolated using a standard protocol, and cDNA was synthesized using M-MLV reverse transcriptase (Promega) following the manufacturer's instructions. One microliter of the synthesized cDNA was used per 20 µl PCR reaction, which comprised of 0.5 U *Taq* DNA polymerase, 1× buffer and 1 mM dNTP mix (Takara) with specific primer pairs, and was amplified as follows; 94°C for 5 min, then 25∼40 cycles of 94°C for 45 s, 56°C for 45 s and 72°C for 1 min, followed by a final extension of 7 min at 72°C using GeneAmp PCR system 2700 (Applied Biosystems, Foster city, CA, USA). PCR primers were designed using the Primer3 program and were purchased from Bioneer (Daejeon, Korea). Sequences of used primers are as follow: IFN-γ forword, 5′- TCCCATGGGTTGTGTGTTTA-3′, reverse, 5′- GAAGCACCAGGCATGAAATC-3′; and GAPDH forward, 5′-CCATCACCATCTTCCAGGAG-3′, reverse, 5′-ACAGTCTTCTGGGTGGCAGT-3′. The PCR products were separated on 1.5% agarose gel, stained with ethidium bromide, visualized by Gel Doc 2000 UV trans-illuminator (Bio-Rad Laboratories, Hercules, CA, USA), and analyzed using Quantity One software (Bio-Rad Laboratories). Each sample was tested more than three times and representative data were showed.

### Confocal microscopy

Cells were cultured on gelatin-coated coverslips, rinsed three times in cold PBS, fixed with 4% paraformaldehyde at room temperature for 20 min, and permeabilized with 0.1% Triton X-100 in PBS for 10 min. The cells were blocked with 1% BSA in PBS for 30 min and then stained with anti-p65 mAbs for 2 hr. Finally, the coverslips were incubated with FITC conjugated rabbit anti-mouse IgG (Molecular Probe, Invitrogen) in dark for 1 hr and the nuclei were stained with 0.1µg/ml DAPI for 5 min. The coverslips containing the cells were mounted on glass slides using VectaShield mounting medium (Vector Laboratories, Burlingame, CA, USA). Confocal images were captured using a Zeiss LSM510META system (Carl Zeiss, Jena, Germany) at 60× magnification with Zeiss LSM Image Browser program. The images were processed and analyzed by the same software.

### Statistical Analysis

All data are the means 6 standard deviations (SDs) of at least 3 independent experiments. Statistical analyses were performed by Student *t* test for data involving two groups or by analysis of variance for data involving more than two groups. P <0.05 was considered statistically significant.

## Results

### GD-1 induces IFN-γ transcription in NK-92 cells

**Figure 1:**
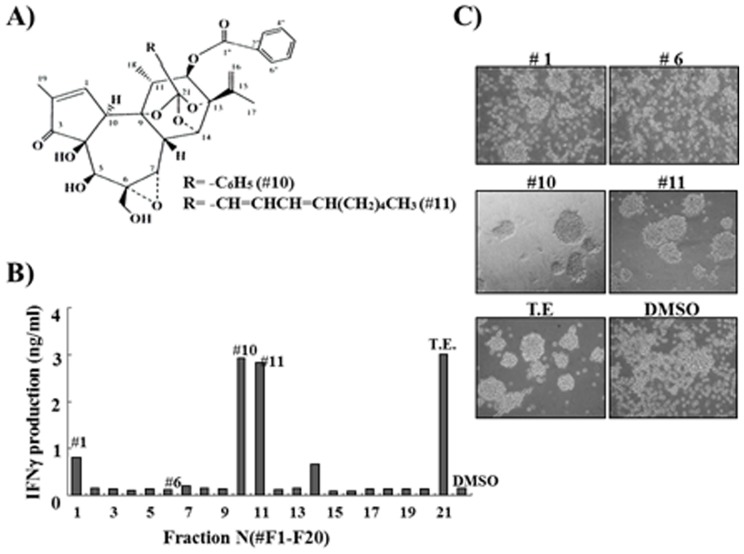
The effects of the flower bud extracts on IFN-γ production and morphological changes. A . The structure of GD-1 and yuanhuacine isolated from the extract of *Daphne genkwa* plant. **B**. ELISA-based assay for IFN-γ production. Total extract (TE) and twenty fractions (1 to 20) were treated on NK-92 and IFN-γ secretion was analyzed by ELISA. Fraction number 10 (#10) and 11 (#11) indicate GD-1 and yuanhuacine, respectively. All ELISA data are representative of at least three independent experiments. **C**. NK-92 cell morphologies were analyzed microscopically after the treatment of flower extracts. The addition of GD-1 (#10), yuanhuacine (#11) and TE have induced the morphological changes of NK-92 cells, which matches with the pattern of IFN-γ production.

Two daphnane diterpene esters has been isolated form the flower buds of *Daphne genkwa*, GD-1 and yuanhuacine, with pro-apoptotic effects (Fig. 1A, [5]). To study the immunogenic potential of the extracts, NK-92 cells were treated with the fractions from the flower bud extracts and the production of immunogenic cytokine, IFN-γ, was analyzed (Fig. 1B). Total extract (TE) and fractions from *Daphne genkwa* plant induced the increase of IFN-γ secretion. When treated with 2 ng of total extract, 3–4 ng/ml of IFN-γ was secreted. Interestingly, the treatment of single fraction, GD-1 (fraction #10) or yuanhuacine (fraction #11), was able to induce of IFN-γ production, similar to the level induced by the treatment of total extracts from the *Daphne genkwa* plant (Fig. 1B). Morphological changes were also induced by the treatment of the extracts as shown in Fig. 1C. Cell aggregation was observed in the GD-1 treated NK-92 cells suggesting that GD-1 might be associated with the modulation of several cytoskeletons and cell adhesion molecules. In fact, modulation of ICAM-1 and alpha-tubulin was observed in GD-1-treated NK-92 cells (data not shown). The expression of ICAM-1 could be regulated by IFN-γ activities [35]. To test the specificity of GD-1 in relation to the induction of IFN-γ production, several human myeloid cell types, U937, HL60, THP-1, and K562, were cultured under the same conditions as NK-92 cells and treated with GD-1. Unlike NK-92 cells, any of the human myeloid cell lines tested did not induce changes in the secretion of IFN-γ by GD-1 treatment (S1A Figure in S1 File). The effect of GD-1 on the production of IFN-γ was superior compared with the other stimuli such as CD40, LPS, and TNFα (S1B Figure in S1 File) without cytotoxicity as determined by WST-1 assay (S2A Figure in S1 File). GD-1-treated NK-92 cells also released IL-10, but not IL-12, IL-2, and TNFα (S3 Figure in S1 File). The increase of IFN-γ secretion by GD-1 treatment seemed to be IL-2 independent because the change of IFN-γ secretion was even occurred under serum-free conditions (S4 Figure in S1 File).

**Figure 2:**
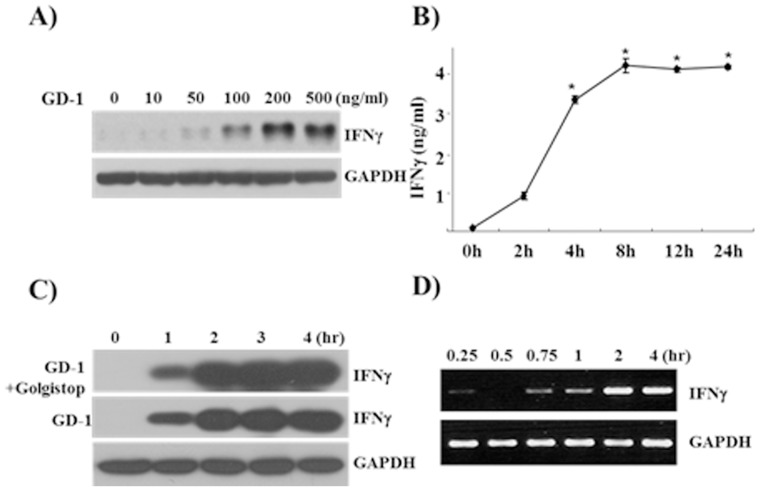
GD-1 regulates the transcription of IFN-γ. **A**. The effect of GD-1 on IFN-γ production was analyzed by Western blotting of the culture medium after GD-1 treatment at various concentrations (0∼200 ng/ml). The production of IFN-γ increased with the increase of GD-1 concentration. **B**. The time-dependent increase of IFN-γ secretion was analyzed by ELISA, which showed the production of IFN-γ reached plateau around 3-hr treatment of GD-1. Triplicate samples in each time were tested and averaged. Error bars indicate standard deviation. All ELISA data are representative of at least three independent experiments. *P<0.05. **C**. The production of IFN-γ was increased in a time-dependent manner. 100 ng/ml of GD-1 was treated onto NK-92 cells, and the culture supernatant was taken at various time points for Western blot analysis. IFN-γ production was seemed to begin within 1 hr. Golgistop treatment has showed that GD-1 does not affect the secretory pathway for IFN-γ production. **D**. Time dependent transcriptional activity for IFN-γ in NK-92 cell after GD-1 treatment was evaluated by RT-PCR. The transcription of IFN-γ was increased within 1 hr after GD-1 treatment.

Increasing concentration of GD-1 treatment showed the dose-dependence of IFN-γ production in NK-92 cells (Fig. 2A). IFN-γ expression increased with the increment of GD-1 concentration and reached a plateau at 200 ng/ml. To search the optimal induction time, 100 ng/ml of GD-1 was added and IFN-γ production was analyzed at various time points. According to ELISA and Western blot analyses, the production of IFN-γ increased between 2-4 hr after GD-1 treatment (Fig. 2B and 2C). The secretory pathway for IFN-γ production was not seemed to be affected by GD-1 treatment because the addition of Golgistop had no effect on IFN-γ induction. RT-PCR analysis of the GD-1-treated NK-92 cells has showed that GD-1 up-regulates the transcription of IFN-γ within 1-hr treatment (Fig. 2D). GD-1 treatment did not affect the cytotoxicity of NK-92 cells (S2A Figure in S1 File). The expression of Granzyme B and Perforin, which are produced from cytotoxic cells to kill their target cells, was not changed by GD-1 treatment (data not shown).

### IFN-γ production by GD-1 treatment is dependent on NF-κB signaling

**Figure 3:**
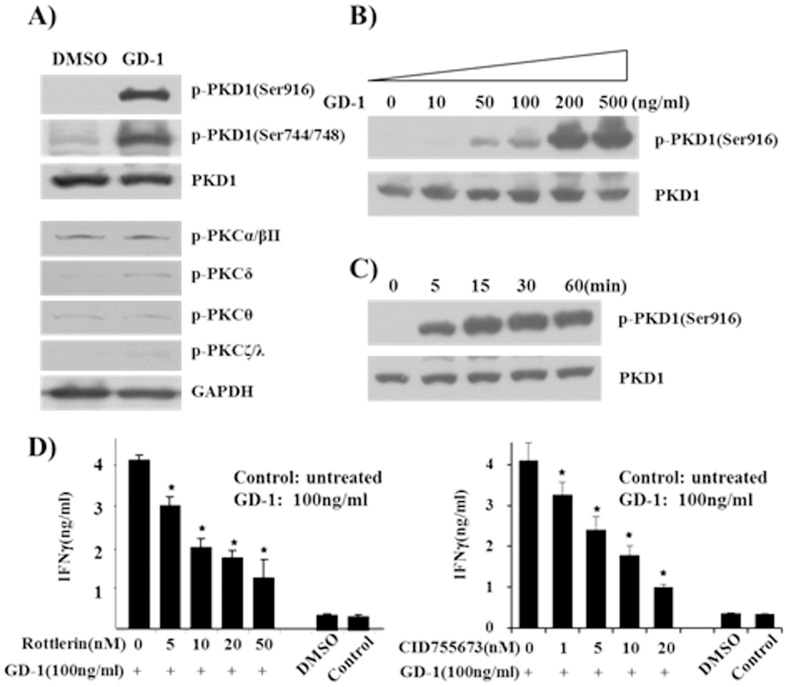
Activation of PKC isoforms in the GD-1 treated NK-92 cells. **A**. 200 ng of GD-1 was treated onto NK-92 cells for 20 min. The cell lysates were analyzed by Western blotting with various antibodies against PKC isoforms, including PKD1. **B**. Phosphorylation of PKD1 was observed at serially diluted GD-1 concentrations with specific antibody to phosphorylated PKD1. **C**. Phosphorylation of PKD1 was observed to increase in a time-dependent manner within 15 min of GD-1 treatment. **D**. The addition of Rottlerin, a PKC inhibitor, and CID755673, a PKD inhibitor, has showed inhibitory effect on GD-1-mediated IFN-γ production, which support the increase of IFN-γ production by GD-1 is dependent of PKC signaling pathway. All ELISA data are representative of at least three independent experiments. Triplicate samples in each time were tested and averaged. Error bars indicate standard deviation. *P<0.05.

Several types of PKC family kinases have been reported to function in the activation of the inflammatory responses by NK cells [29]–[33]. To identify the responsible signaling pathway for the activation of IFN-γ transcription, we have analyzed the phosphorylation status of PKC family members and found the phosphorylation of protein kinase D1 (PKD1) was increased in NK-92 cells treated with GD-1 (Fig. 3). PKD1, which also called PKCδ1 or PKCµ, is a member of PKC protein kinase family. As shown in Fig. 3A, the exposure of NK-92 cells to GD-1 for 20 min led to a significant increase of phosphorylated PKD1, but not other PKC isoforms. All the phosphorylation sites analyzed, Ser916 and Ser744/748, were shown to be phosphorylated by GD-1 treatment. GD-1 was applied to NK-92 cells in a range from 0 to 200 ng/ml for 20 min, and the level of phospho-PKD1 (Ser916) was compared (Fig. 3B). GD-1 caused the increase of PKD1 phosphorylation in a dose-dependent manner with saturation at 200 ng/ml. In addition, PKD1 phosphorylation started immediately after GD-1 treatment and increased for 15 min (Fig. 3C). These results suggest that GD-1 controls the phosphorylation of PKD1 in NK-92 cells in dose- and time-dependent manners. Also, Rottlerin a PKC inhibitor, and CID755673, a PKD inhibitor [36], have inhibited GD-1-induced IFN-γ production (Fig. 3D). From these data, we have confirmed that PKD1 activity is a critical role for the regulation of IFN-γ production by GD-1 treatment in NK-92 cells.

**Figure 4:**
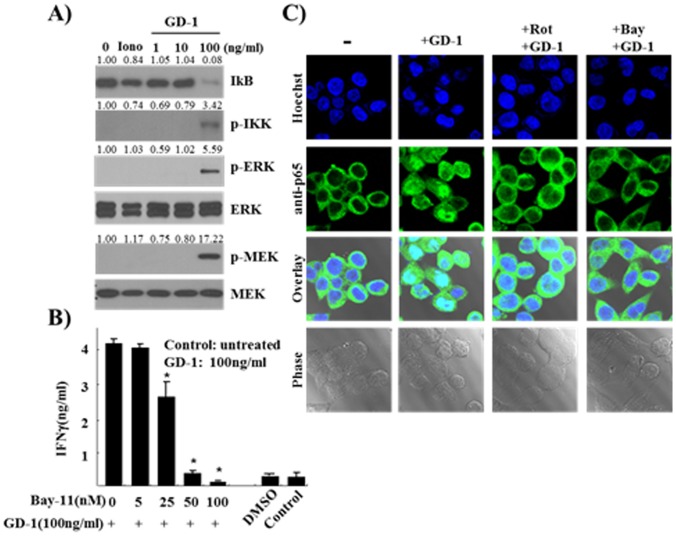
IFN-γ production induced by GD-1 is dependent on NF-κB activity. **A**. GD-1 was treated onto NK-92 cells at 1 to 100 ng/ml concentrations, and several down-stream targets for PKC signaling were analyzed by Western blotting. The phosphorylation of IKK was detected with the concomitant degradation of IκB. ERK and MEK were also phosphorylated by GD-1 treatment. The bands were quantified using Quantity One 1-D Analysis Software (Bio-Rad Laboratories) and p-IKK and IκB expression normalized to control their protein expression. And p-ERK and p-MEK expressions normalized to total their protein expression. The values are given above the blots. **B**. Inhibitors of NF-κB, Bay11-7082, was treated with a serial dilution from 100 µM with 10 0 ng/ml GD-1, and IFN-γ production was analyzed by ELISA. The addition of Bay11-7082 has showed an inhibitory effect on GD-1 activity to increase IFN-γ production. All ELISA data are representative of at least three independent experiments. Triplicate samples in each time were tested and averaged. Error bars indicate standard deviation. *P<0.05. **C**. The nuclear translocation of p65 (an NF-κB subunit) was observed in GD-1-stimulated NK-92 cells. The modulation of cytoskeleton was observed through Phase contrast images and DAPI was used to visualize nuclei. GD-1 treatment onto NK-92 cells induced nuclear localization of cytosolic p65 transcription factor, which was inhibited by the co-addition of Rottlerin or Bay11-7082.

Multiple MAPK signaling pathways are involved in the regulation of IFN-γ production from the stimulated NK cells [37]. PKC signaling can activate MAPK pathways via Ras-dependent or -independent pathway. To see downstream effector molecule(s) for the activation of IFN-γ expression, several kinase inhibitors including Wortmannin (PI3K inhibitor), S203580 (p38 MAPK inhibitor), PD 98059 (MAPK kinase inhibitor), LY27632 (ROCK inhibitor), and ZM336372 (Raf kinase inhibitor) were added to the GD-1-treated NK-92 cells. However, any of the inhibitors could not block the induction of IFN-γ secretion by GD-1 treatment although the phosphorylation of ERK and MEK were increased (Fig. 4A). Our data suggests that ERK/MEK signaling pathway can be activated by GD-1 treatment probably via PKC/PKD signaling [38], but their activation is not related with the regulation of IFN-γ expression. Among the kinases tested, the phosphorylation of IKK was increased by GD-1 treatment followed by the degradation of IκB, which could cause nuclear translocation of NF-κB subunits to activate their target gene expression (Fig. 4A).

NF-κB has been reported to function on the promoter region of IFN-γ gene to regulate its transcription [39]. To demonstrate that the production of IFN-γ is dependent on NF-κB signaling pathway, NF-κB inhibitors, including Bay11-7082, CAPE, and PDTC, were treated on NK-92 cells cultured with GD-1. As shown in Fig. 4B, IFN-γ production was decreased by the treatment of Bay11-7082 in a concentration-dependent manner. The nuclear translocation of p65, one of NF-κB subunit, was induced by GD-1 treatment (Fig. 4C). Although precise localization was difficult because of the small cytosol in this NK cells, more portions of p65 protein were colocalized with DAPI nuclear stain. The addition of Rottlerin and Bay11-7082 inhibited the nuclear localization of p65 protein empathizing the involvement of PKC and NF-κB signaling.

These data suggest that the induction of IFN-γ by GD-1 is mediated by PKD1-mediated NF-κB signaling pathway. Both PKC and ERK/MEK pathways could activate NF-κB transcriptional activities by phosphorylating IKKs via separate pathways [40], [41]. Because the treatment of ERK/MEK inhibitors could not inhibit the effect of GD-1 on IFN-γ production (data not shown), NF-κB activation by GD-1 treatment could be mediated through the IKK phosphorylation by PKD1 via MAPK-independent pathway. The exact biological mechanism of GD-1 in the production of IFN-γ remains to be clarified.

### STAT1 functions as a positive feedback for IFN-γ production

**Figure 5:**
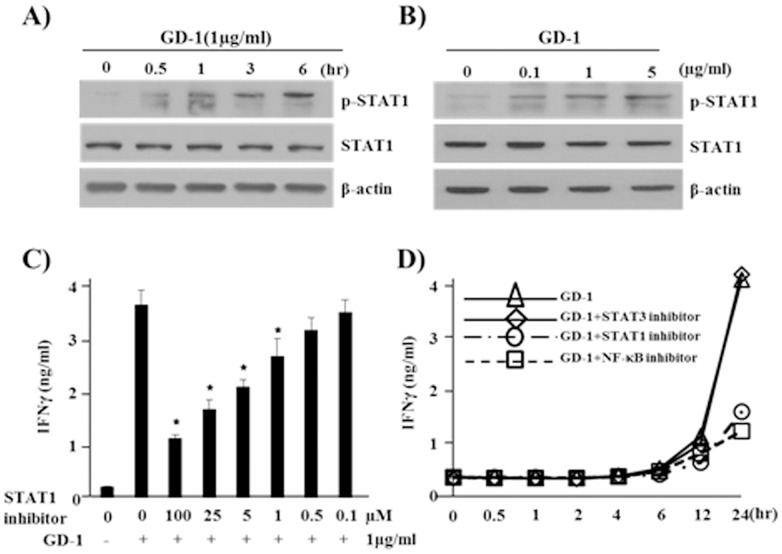
STAT1 signaling pathway is activated by GD-1 treatment. **A**. The phosphorylation of STAT1 was induced within 1 hr after GD-1 treatment. **B**. The increase of phosphorylated STAT1 was detected at high concentration of GD-1 (>1 µg/ml). **C**. The addition of STAT1 inhibitor blocked IFN-γ production induced by GD-1 treatment on a concentration-dependent manner. All ELISA data are representative of at least three independent experiments. Triplicate samples in each time were tested and averaged. Error bars indicate standard deviation. *P<0.05. **D**. The induction of IFN-γ production in GD-1-treated NK-92 cells was inhibited by the addition of STAT1 and NF-κB inhibitors but by STAT3 inhibitor.

STAT1 is a well-known downstream target of IFN-γ activation [42]. The phosphorylation of STAT1 was increased by the treatment of GD-1 reaching plateau around 1-hr (Fig. 5A, B). Surprisingly, the addition of STAT1 inhibitor could induce the decrease of IFN-γ production from GD-1-treated NK-92 cells (Fig. 5C), which means STAT1 activity is required for the expression of IFN-γ as a positive feedback mechanism. STAT3 inhibitor did not affect IFN-γ production suggesting the specific effects of STAT1 and NF-κB pathways on IFN-γ production (Fig. 5D).

## Discussion

In this study, to isolate NK cell activators in the natural compounds originated from plant extracts, we used ELISA kits to evaluate secreted cytokines, including IFN-γ, in the culture supernatant of NK-92 cells treated with the extracts. As a result, the extracts of *Daphne genkwa* were found to induce IFN-γ production, and GD-1 and yuanhuacine from the extracts were purified and characterized to be the functional fractions. Here, the function and biological roles of GD-1 and yuanhuacine on NK cells have been discovered as an inducer of IFN-γ secretion.

NK cells are critical effector cells providing a primary defense mechanism of innate immune response prior to the initiation of adaptive immune response. The major functions of NK cells are cytotoxicity and cytokine production on target cells by activating their receptors against foreign ligands. Both arms of the NK cell effector functions participate in both the direct innate defense and the shaping of the adaptive immune response. In several mouse models, NK cells have been shown to limit the development of tumors and combat microbial infections. In particular, NK cells control the early steps of mouse cytomegalovirus (MCMV) infection, both by directly killing virus-infected cells and by producing IFN-γ [7]. NK-specific cytotoxic receptors, which called natural cytotoxic receptors (NCRs), include NKp46 [8], NKp30 [9], and NKp44 [10]. Hemagglutinin (HA) of influenza virus (IV), hemagglutinin-neuraminidase (HN) of Sendai virus (SV) [11], and membrane-associated heparin sulfate proteoglycans [12] are known ligands of NCRs.

IFN-γ is classified as a type II interferon produced by the activated T and NK cells of the immune system of most vertebrates in response to challenges by foreign agents such as viruses, parasites and tumor cells [9], [10]. The primary sources of IFN-γ are NK cells and NKT cells, which are effectors of the innate immune response, and also CD8 and CD4 Th1 effector T cells of the adaptive immune system. IFN-γ has long been recognized as a signature proinflammatory cytokine that plays a central role in inflammation and autoimmune disease. There is now emerging evidence indicating that IFN-γ possesses unexpected properties as a master regulator of immune responses and inflammation. IFN-γ is typically produced by NK and T cells in response to foreign agents such as bacteria, including *Francisella tularensis* [43], viruses such as human cytomegalovirus [44] and influenza virus [45], and parasites such as *Leishmania* [46]. As intrinsic factors, IL-2 [47], IL-12 [48], IL-15, IL-18 [49], and IL-21 [50] are known to be the primary cytokines along with the production of IFN-γ by NK cells. We also observed an induction in the production of IFN-γ with IL-15 comparing PMA and GD-1. GD-1 was more effective in producing IFN-γ than IL-15. Production of IFN-γ with IL-15 treatment also was reduced by Bay11-7082, IKKα inhibitor, which is due to the NF-kB inhibition. One well known agent for IFN-γ production in NK cell is PMA/Ionomycine [51]. Our data suggest that GD-1 is a novel IFN-γ inducer. A small amount (2 ng/ml) of GD-1 efficiently induces IFN-γ in NK-92 cells (S1A Figure in S1 File) as well as in vitro differentiated NK cells (data not shown). GD-1 induces higher amounts of IFN-γ than LPS, CD40 or TNFα (S1B Figure in S1 File).

It was reported that the activation of PKCθ is required for IFN-γ production in response to high concentration of IL-12 [29]. Here, we found that the activation of PKD1 was required for IFN-γ secretion in GD-1 treated NK-92 cells. Phosphorylation of PKD1 activates IKK beta phosphorylation, leading to IκB degradation and subsequent p65 and p50 translocation into the nucleus, ultimately activating the transcriptional activity of NF-κB [52]. Our data show that the phosphorylation of PKD1 was dose- and time-dependently increased by GD-1 treatment on NK-92 cells (Fig. 3). Furthermore, downstream targets of the PKC pathway such as ERK, MEK and IKK were phosphorylated, which resulted in the degradation of IκB. In addition to the role of GD-1 as a NF-κB signaling activator, the expression level of the NF-κB subunits, p50 and p65, was investigated by RT-PCR and Western blot analysis in the GD-1-treated NK-92 cells (data not shown).

The addition of kinase inhibitors, Rottlerin for PKC inhibition or Bay11-7082 for IKK inhibition, has supported the role of GD-1 in NF-κB activation, which blocked the nuclear translocation of p65 and the production of IFN-γ. In summary, it was showed that GD-1 has a potent effect on the IFN-γ production in NK-92 cell lines. IFN-γ production in NK cells by treatment of GD-1 is mediated by the activation of NF-κB and an up-regulated expression of p50 and p65. These finding suggest that GD-1 might be an attractive target as an immune modulator with potential to be used as a therapeutic anti-viral drug.

## Supporting Information

S1 File Cytotoxicity, optimum culture conditions and concentrations of GD-1 on IFN-γ production in NK-92 cells. **S1 Figure**, Determination of effective concentration of GD-1 for IFN-γ production in human myeloid cell lines and comparison between GD-1 and other stimuli, CD40, LPS, TNFα on IFN-γ release from NK-92 cell. **A**. Evaluation of IFN**-γ** production in the human myeloid cell lines, U937, HL-60, THP-1, K562, and NK-92. All ELISA data are representative of at least three independent experiments. **B**. Evaluation of IFN**-γ** production in the NK-92 cell line in presence of immune stimuli, CD40 (1∶10 ng/ml, 2∶1 ng/ml), LPS (1:100 ng/ml, 2∶10 ng/ml), TNFα (1∶5 ng/ml, 2∶0.5 ng/ml) and GD-1 (1∶2µg/ml, 2∶200 ng/ml). All ELISA data are representative of at least three independent experiments. Triplicate samples in each time were tested and averaged. Error bars indicate standard deviation. *P<0.05. **S2 Figure**, Cytotoxicity of GD-1 during IFN-γ production under GD-1 treated NK-92 cell. **A.** Cell proliferation was measured using the WST-1 reagent (Roche Applied Science) according to the manufacturer's protocol. NK-92 cells were seeded about 2x10^4^ cells per well in 100µl media volume on a 96 well flat-bottom plate. After seeding cells, wells were treated with the indicated concentration of GD-1 for 12hr. GD-1 have no significant cytotoxicity on the NK-92 in the dose range from 0 to 200 ng/ml. **B.** NK-92 cell was treated GD-1 under condition of serial concentrations from 0 to 200 ng/ml for 12hr. IFN-γ production by GD-1(100 ng/ml) in culture supernatant was saturated. All ELISA data are representative of at least three independent experiments. Triplicate samples in each time were tested and averaged. Error bars indicate standard deviation. *P<0.05. **S3 Figure**, Evaluation of secreted cytokines production in the GD-1 treated human myeloid cells. The secreted IL-12 (**A**), IL-2 (**B**), TNFα (**C**) and IL-10 (**D**) were examined in the GD-1 treated NK-92 cells. All ELISA data are representative of at least three independent experiments. Triplicate samples in each time were tested and averaged. Error bars indicate standard deviation. *P<0.05. **S4 Figure**, Optimum culture conditions for IFN-γ production under GD-1 treated NK-92 cells. GD-1-treated NK-92 cells were cultured under condition of a serial concentration of FCS (from 20% to 0%) and with (+) or without (-) of IL-2 for 12hr. All ELISA data are representative of at least three independent experiments. Triplicate samples in each time were tested and averaged. Error bars indicate standard deviation. *P<0.05.
(DOCX)
