# Supplementary material for: Genkwadaphnin Induces IFN-γ via PKD1/NF-κB/STAT1 Dependent Pathway in NK-92 Cells
Source: PLoS One. 2014 Dec 17;9(12):e115146. doi: 10.1371/journal.pone.0115146 (PMC4269520; doi:10.1371/journal.pone.0115146)

**Supplementary Data**

**Genkwadaphnin induces IFN-γ via PKD1/NF-κB/STAT1 dependent pathway in NK-92 cells**

**Ho-Bum Kang, Kyung-Seop Ahn,** **Sei-Ryang Oh and Jae Wha Kim^*^**

**Requests for reprints**: Jae Wha Kim, Ph. D., Medical Genomics Research Center, Korea Research Institute of Bioscience and Biotechnology, Daejeon 305-333, Republic of Korea. Tel: +82-42-860-4238, Fax: +82-42-860-4593 E-Mail: [wjkim@kribb.re.kr](mailto:wjkim@kribb.re.kr).

Figure S1





Figure S2





Figure S3





Figure S4


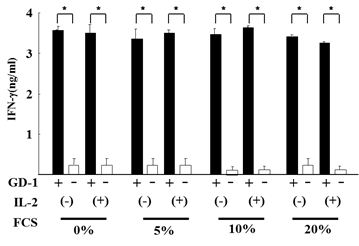

Supplement: S1 File — Cytotoxicity, optimum culture conditions and concentrations of GD-1 on IFN-γ production in NK-92 cells. S1 Figure, Determination of effective concentration of GD-1 for IFN-γ production in human myeloid cell lines and comparison between GD-1 and other stimuli, CD40, LPS, TNFα on IFN-γ release from NK-92 cell. A. Evaluation of IFN-γ production in the human myeloid cell lines, U937, HL-60, THP-1, K562, and NK-92. All ELISA data are representative of at least three independent experiments. B. Evaluation of IFN-γ production in the NK-92 cell line in presence of immune stimuli, CD40 (1∶10 ng/ml, 2∶1 ng/ml), LPS (1:100 ng/ml, 2∶10 ng/ml), TNFα (1∶5 ng/ml, 2∶0.5 ng/ml) and GD-1 (1∶2µg/ml, 2∶200 ng/ml). All ELISA data are representative of at least three independent experiments. Triplicate samples in each time were tested and averaged. Error bars indicate standard deviation. *P<0.05. S2 Figure, Cytotoxicity of GD-1 during IFN-γ production under GD-1 treated NK-92 cell. A. Cell proliferation was measured using the WST-1 reagent (Roche Applied Science) according to the manufacturer's protocol. NK-92 cells were seeded about 2x10^4^ cells per well in 100µl media volume on a 96 well flat-bottom plate. After seeding cells, wells were treated with the indicated concentration of GD-1 for 12hr. GD-1 have no significant cytotoxicity on the NK-92 in the dose range from 0 to 200 ng/ml. B. NK-92 cell was treated GD-1 under condition of serial concentrations from 0 to 200 ng/ml for 12hr. IFN-γ production by GD-1(100 ng/ml) in culture supernatant was saturated. All ELISA data are representative of at least three independent experiments. Triplicate samples in each time were tested and averaged. Error bars indicate standard deviation. *P<0.05. S3 Figure, Evaluation of secreted cytokines production in the GD-1 treated human myeloid cells. The secreted IL-12 (A), IL-2 (B), TNFα (C) and IL-10 (D) were examined in the GD-1 treated NK-92 cells. All ELISA data are representative of at least three in [file pone.0115146.s001.docx]
